# Silence of Long Noncoding RNA SNHG14 Alleviates Ischemia/Reperfusion-Induced Acute Kidney Injury by Regulating miR-124-3p/MMP2 Axis

**DOI:** 10.1155/2021/8884438

**Published:** 2021-01-04

**Authors:** Qianlong Xue, Lipeng Yang, Hui Wang, Shuchi Han

**Affiliations:** Department of Emergency, The First Affiliated Hospital of Hebei North University, No. 12, Changqing Road, Zhangjiakou City, Hebei Province 075000, China

## Abstract

**Purpose:**

Ample evidence has proved that lncRNAs are pivotal regulators in acute kidney injury (AKI). Here, we focus on the role and mechanism of lncRNA SNHG14 in ischemia/reperfusion- (I/R-) caused AKI.

**Methods:**

I/R and hypoxia/reoxygenation (H/R) were applied to induce rats and HK-2 cells to establish AKI models in vivo and in vitro. Relative expression of SNHG14, miR-124-3p, and MMP2 was determined by qRT-PCR. HE staining was used to evaluate pathological changes in renal tissues, and acute tubular necrosis (ATN) score was calculated. Renal function was evaluated by measuring serum creatinine content and blood urea nitrogen content. Levels of IL-1*β*, IL-6, and TNF-*α* were measured by ELISA. Cell viability was examined by MTT assay. Oxidative stress was assessed by measuring SOD, MDA, and ROS levels. The target of SNHG14 or miR-124-3p was verified by DLR assay. Protein expression of MMP2 was examined by western blot.

**Results:**

SNHG14 was boosted in renal tissues of I/R-stimulated rats and H/R-induced HK-2 cells, while miR-124-3p was diminished in H/R-stimulated HK-2 cells. Si-SNHG14 or miR-124-3p mimics repressed inflammation and oxidative stress and enhanced cell viability in H/R-stimulated HK-2 cells. Sh-SNHG14 mitigated I/R-induced AKI in rats. MiR-124-3p was targeted by SNHG14, and MMP2 was targeted by miR-124-3p. Inhibition of miR-124-3p or upregulation of MMP2 reversed inhibitory effects of SNHG14 silence on inflammation and oxidative stress as well as the promoting effect of SNHG14 silence on cell viability in H/R-induced HK-2 cells.

**Conclusion:**

Knockdown of SNHG14 alleviated I/R-induced AKI by miR-124-3p-mediated downregulation of MMP2.

## 1. Introduction

Acute kidney injury (AKI) is defined as a sudden decline of kidney function [1]. Excessive production of inflammatory cytokines, necrosis, apoptosis, and delayed proliferation of renal resident cells are major pathological features of AKI [2–4]. As a common contributor of AKI [5], renal ischemia-reperfusion (I/R) injury leads to injury of different degrees to renal tissues, which is related to the increment of morbidity and mortality [6–8]. It arises from momentary interruption of blood supply followed by restoring blood supply, which fails to restore renal functions and causes structural damage [9, 10]. Currently, renal replacement treatment and dialysis are the main strategies of AKI therapy [11, 12]. Despite several clinical advancements, the therapeutic effect is still unsatisfactory [13, 14]. Therefore, exploring a new strategy to alleviate I/R-induced renal injury is urgent and imperative for developing effective treatments of AKI.

Long noncoding RNAs (lncRNAs) are a type of nonprotein-coding RNAs with a length of more than 200 nucleotides [15, 16]. Mounting evidence has certified that lncRNAs are critical molecular mediators in regulating the pathological progression of I/R-induced renal injury [17–19]. Xu et al. have stated that silencing of lncRNA TUG1 mitigates I/R-induced apoptosis and inflammation response in renal tubular epithelial cells [17]. Xie et al. have declared that downregulation of lncRNA LINC00963 alleviates I/R-caused renal injury in human kidney proximal tubular cells (HK-2 cells) [18]. Tian et al. have revealed that silence of lncRNA LINC00520 lightens renal injury caused by I/R in rats and represses oxidative stress in hypoxia/reoxygenation- (H/R-) stimulated HK-2 cells [19]. It is noteworthy that small nucleolar RNA host gene 14 (SNHG14), a novel lncRNA mapping to 15q11.2 in humans [20], has been uncovered to make imperative impacts on diverse diseases [21, 22]. One observation from Liu et al. has denoted that upregulation of lncRNA SNHG14 facilitates cell invasion and migration in renal cell carcinoma [21]. Moreover, a recent study has unveiled that knockdown of lncRNA SNHG14 reduces inflammatory response caused by cerebral I/R injury [22]. However, the role and mechanism of SNHG14 in I/R-induced AKI have not been expounded till now.

MicroRNAs (miRNAs), a sort of small intracellular molecules with a length of 18-23 nucleotides, can suppress the expression of genes through affecting mRNA degradation or repressing translation [23]. And, numerous miRNAs have been disclosed to participate in the progression of I/R-induced renal injury [24]. For example, suppression of miR-377 is reported to repress I/R-triggered renal inflammation and oxidative stress in mice [25]. MiR-126 is validated to suppress oxidative stress by increasing superoxide dismutase (SOD) and decreasing malondialdehyde (MDA) in I/R-induced renal models of mice [26]. As a widely explored miRNA, miR-124-3p has been documented to exert critical roles in I/R-caused injury [27, 28]. Liang et al. have indicated that upregulation of miR-124-3p reduces H/R-caused production of tumor necrosis factor-alpha (TNF-*α*), interleukin-1 beta (IL-1*β*), and IL-6 in human cardiac myocytes [27]. Li et al. have declared that increasing miR-124-3p attenuates I/R-induced nerve injury in the spinal cord [28]. Nonetheless, the modulation of miR-124-3p by SNHG14 in I/R-induced renal injury has not been clarified.

Matrix metalloproteinase 2 (MMP2), also known as gelatinase A, is widely found in somatic tissues and responsible for the degradation of extracellular matrix (ECM) [29, 30]. Abundant studies have revealed the involvement of MMP2 in I/R-induced injury [17, 31, 32]. Li et al. have reported that silencing of MMP2 attenuates the inhibitory effect of miR-125 inhibitor on I/R-induced acute myocardial infarction by promoting oxidative stress [31]. Zeng et al. have revealed that morroniside protects against cerebral I/R injury by suppressing MMP2/9 expression [32]. Importantly, a previous study has indicated that silencing of TUG1 attenuates H/R-induced inflammation and apoptosis of renal tubular epithelial cells via sponging miR-449b-5p to regulate MMP2 expression [17]. MMP2 is reported to act as the downstream target of many miRNAs, such as miR-29a [33], miR-125 [31], and miR-2681 [34]. However, the regulatory relationship between miR-124-3p and MMP2 and the regulatory mechanism of SNHG14/miR-124-3p/MMP2 axis in I/R-induced AKI have not been explored.

The objective of the current study was to elucidate the expression and function of SNHG14 in I/R-induced rat model and H/R-induced cell model. Additionally, to illustrate the molecular mechanism of SNHG14 in I/R-induced AKI, the interrelation among SNHG14, miR-124-3p, and MMP2 was investigated.

## 2. Methods

### 2.1. Construction of Renal Ischemia/Reperfusion (IR) Models

Male Sprague Dawley rats (10-week-old, SPF) were bought from Shanghai Animal Laboratory Center (Shanghai, China). Rats were reared in a controlled environment with a 12 h light/dark cycle, a humidity of 60%, and a temperature of 21-24°C and had free access to food and water. This research was supported by the Animal Care and Use Committee of our hospital, and animal experiments were operated in accordance with the National Institutes of Health Guide for the Care and Use of Laboratory Animals.

Forty rats were randomly assigned into four groups (*n* = 10/group): the sham group, the I/R group, the short hairpin- (sh-) negative control (NC) group, and the sh-SNHG14 group. Sh-SNHG14 or sh-NC (RiboBio, Beijing, China) was introduced into the adenoviral vector (LifeTechnologies, Shanghai, China) to form adenovirus solution of sh-SNHG14 or sh-NC by using Gateway™ LR Clonase IIEnzyme Mix (Invitrogen, Carlsbad, CA, USA) as previously stated [35]. A week before I/R operation, adenovirus solution of sh-NC or sh-SNHG14 (20 *μ*L, 10^7^ particles/*μ*L) was delivered into the tail vein of rats in the sh-NC or sh-SNHG14 group [36].

To induce renal I/R models in vivo, rats were firstly anesthetized through intraperitoneal injection of sodium pentobarbital (50 mg/kg). Next, kidneys were exposed by midline laparotomy. The right kidney was resected, and the left renal pedicle was occluded with nontraumatic clamps for 45 min, followed by a 24 h reperfusion as previously presented [37, 38]. Occlusion was affirmed visually via a color change. After clamps being removed, recovery of blood flow was observed. Rats in the sham group received the same operations apart from the closure of the renal pedicle. After reperfusion for 24 h, all rats were anesthetized by intraperitoneally injecting sodium pentobarbital (50 mg/kg) and then euthanized via cervical dislocation. Renal tissues and blood were collected for the following investigation.

### 2.2. Assessment of Renal Function

For collecting serum, blood samples were centrifuged (3,000 rpm) at 4°C for 20 min. The parameters of renal function including relative serum creatinine (Cr) content and relative urea nitrogen (BUN) content were measured by commercial kits (Jiancheng, Nanjing, China), respectively.

### 2.3. Hematoxylin-Eosin (HE) Staining

The separated renal tissues were fixed in 4% paraformaldehyde at 4°C overnight and paraffin-embedded. The paraffin-embedded sections were cut into slices (5 *μ*m) and then deparaffinized by graded ethanol. After HE staining, xylene and graded ethanol were utilized for dehydration, and the sections were observed via a Nikon Eclipse Ti-S microscope (Nikon, Tokyo, Japan). The pathological injury was quantified using an acute tubular necrosis (ATN) scoring system as previously described [39].

### 2.4. Quantitative Real-Time Polymerase Chain Reaction (qRT-PCR)

Total RNAs in kidney tissues of rats and HK-2 cells were extracted by TRIzol reagent (Thermo Fisher, Waltham, MA, USA) and converted to complementary DNAs (cDNAs) with a SuperScript III Reverse Transcriptase kit (Thermo Fisher). The qRT-PCR was implemented via SYBR Premix Ex Taq II (Takara, Dalian, China). Primers for qRT-PCR were procured from GeneCopoeia (Guangzhou, China), and sequences were manifested in Table 1. Finally, the relative expression of SNHG14 and miR-124-3p was calculated with the 2^-*ΔΔ*Ct^ method and normalized against GAPDH or U6.

### 2.5. Cell Culture and Establishment of H/R Cell Models

HK-2 cells purchased from the Chinese Cell Bank of the Chinese Academy of Sciences (Shanghai, China) were nurtured in Dulbecco's modified Eagle's medium (DMEM) including 10% fetal bovine serum (FBS), 100 *μ*g/mL streptomycin, and 100 U/mL penicillin. All cells were maintained at 37°C in a humidified incubator with 95% air and 5% CO_2_.

To simulate renal H/R models in vitro, HK-2 cells were treated by H/R. In detail, HK-2 cells were firstly cultured in hypoxia conditions containing 1% O_2_, 5% CO_2_, and 94% N_2_ for 24 h, and then, cells were subject to 21% O_2_ for 12 h [40]. The control cells were maintained in normal atmosphere with 95% air and 5% CO_2_.

### 2.6. Cell Transfection

The small interfere- (si-) NC, si-SNHG14, miR-124-3p inhibitor, miR-124-3p mimics, miR-NC, and pcDNA-MMP2 were bought from RiboBio. When HK-2 cells grew 80%-90% confluence, the above transcripts were transfected into HK-2 cells with Lipofectamine 3000 (Invitrogen) for 48 h.

### 2.7. Detection of Reactive Oxygen Species (ROS), MDA, and SOD

The fluorescence probe 2′,7′-dichlorodihydrofluorescein diacetate (DCHF-DA; Jiancheng) was applied to detect relative level of ROS. In brief, HK-2 cells were rinsed by PBS and then stained with DCFH-DA (10 *μ*M) for 30 min under the dark. A fluorescence microscope was utilized for examining the fluorescence of DCFH-DA.

A Lipid Peroxidation MDA Assay Kit and a Total SOD Assay Kit (Beyotime, Jiangsu, China) were used to measure relative levels of MDA and SOD in the supernatant.

### 2.8. Cell Counting Kit-8 (CCK-8) Assay

A CCK-8 proliferation detection kit (Dojindo, Kumamoto, Japan) was employed to gauge cell viability. In short, HK-2 cells were plated into 96-well plates (2,000 cells/well) and maintained at 37°C for 24 h. Then, each well was supplemented with 10 *μ*L CCK-8 (Dojindo) to nurture for additional 2 h. Finally, the optical density was observed at 450 nm through a microplate reader.

### 2.9. Enzyme-Linked Immune Sorbent Assay (ELISA)

Concentrations of IL-1*β*, IL-6, and TNF-*α* in serum of rats and culture supernatant of HK-2 cells were measured using commercial ELISA kits (R&D System, Minneapolis, MN, USA) following manufacturer's recommendations. The optical density was examined at 450 nm with a Power Wave Microplate Reader (Bio-TEK, USA).

### 2.10. Dual-Luciferase Reporter (DLR) Assay

The 3′-UTR fragment of wild-type (wt) SNHG14 or wt MMP2 containing the complementary sequence of miR-124-3p was introduced into a pGL3 Basic Vector (Promega, Madison, WI) to construct SNHG14 wt vector or MMP2 wt vector. In a similar way, the 3′-UTR segment of mutant-type (mut) SNHG14 or mut MMP2 holding the mutant sequence of miR-124-3p was inserted into a pGL3 Basic Vector (Promega) to generate SNHG14 mut vector or MMP2 mut vector. Then, HK-2 cells were cotransfected with a wt vector or mut vector and miR-124-3p mimics or miR-NC. After transfection for 48 h, relative luciferase activity was examined by a Dual-Luciferase Reporter Assay System (Promega).

### 2.11. Western Blot

Proteins from HK-2 cells were dissociated with RIPA lysis buffer (Beyotime, Shanghai, China). Equivalent amounts of proteins were resolved by 10% SDS-PAGE gels and blotted on polyvinylidene fluoride (PVDF) membranes. Subsequently, the membranes were blocked with 5% skim milk and then nurtured with the following primary antibodies (Abcam, Cambridge, UK) at 4°C overnight: anti-MMP2 (1 : 2000, ab92536, Abcam) and anti-*α*-tubulin (1 : 2000, ab52866, Abcam). After being washed with tris-buffered saline Tween (TBST), the membranes were incubated with the secondary antibody (anti-rabbit) at 37°C for 2 h. A Chemiluminescence Detection Kit (Thermo Fisher) was used for visualization of immune reactivity. Relative protein expression of MMP2 was quantified through Alphalmager™ 2000 Imaging System (Alpha Innotech, San Leandro, USA) and normalized based on the gray value of *α*-tubulin.

### 2.12. Statistical Analysis

The SPSS 22.0 software was utilized for statistical analyses. Data from three independent experiments were illustrated as the mean ± standard deviation. Differences between two groups were analyzed by Student's *t*-test. The comparisons among more than two groups were assessed via one-way ANOVA followed by Turkey's post hoc test. *P* < 0.05 was considered to be statistically significant.

## 3. Results

### 3.1. SNHG14 Was Upregulated in Renal Tissues of I/R-Induced Rats, and Its Knockdown Alleviated I/R-Induced Renal Injury in Rats

**Figure 1:**
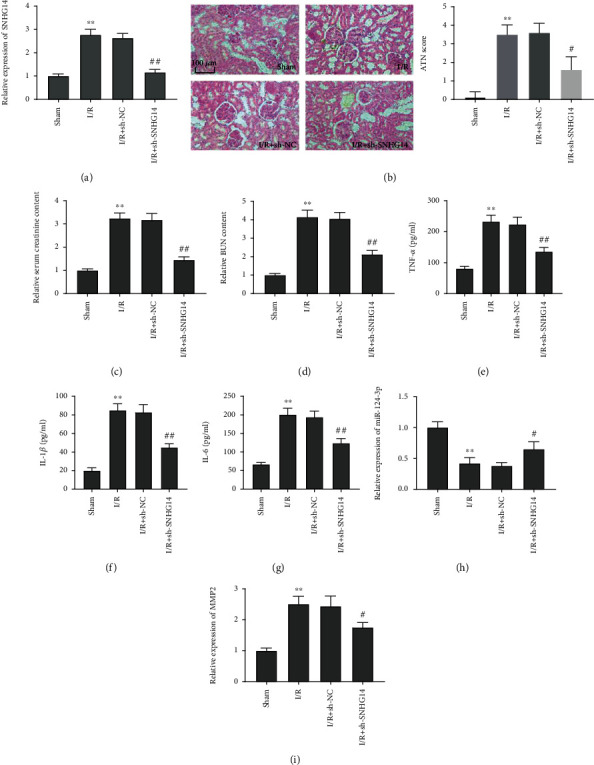
SNHG14 knockdown alleviated I/R-induced renal injury in rats. (a) Relative expression of SNHG14 in renal tissues. (b) HE staining of renal tissues (magnification 400x, scale bar = 100 *μ*m) and kidney acute tubular necrosis (ATN) score. (c) Serum creatinine. (d) Blood urea nitrogen (BUN). (e) Serum TNF-*α* level. (f) Serum IL-1*β* level. (g) Serum IL-6 level. (h) Relative expression of miR-124-3p in renal tissues. (i) Relative expression of MMP2 in renal tissues. ^∗∗^*P* < 0.01, vs. sham. ^#^*P* < 0.05, ^##^*P* < 0.01, vs. I/R+sh-NC.

For purpose of exploring the function of SNHG14 in I/R-induced rats, we initially determined the relative expression of SNHG14 by qRT-PCR in renal tissues. As displayed in Figure 1(a), SNHG14 expression in the I/R group was markedly elevated as opposed to the sham group, and transfection of sh-SNHG14 abolished the elevation of SNHG14 induced by I/R in renal tissues of rats (all *P* < 0.01, Figure 1(a)). Subsequently, we probed the specific role of SNHG14 in I/R-induced rats. Obvious renal injuries comprising cell necrosis, tubular dilatation, and cast formation were observed in I/R-induced rats, and knockdown of SNHG14 attenuated these pathological changes to some degrees. The ATN score was significantly higher in the I/R group than that in the sham group (*P* < 0.01), and the transfection of sh-SNHG14 reversed I/R-induced elevation of ATN score (*P* < 0.05, Figure 1(b)). In addition, I/R treatment contributed to the increases of relative serum Cr content and relative BUN content, which was reversed by the addition of sh-SNHG14 in serum of I/R-induced rats (all *P* < 0.01, Figures 1(c) and 1(d)). Additionally, notable increment of inflammatory factors (IL-6, IL-1*β*, and TNF-*α*) was observed in the I/R group compared with the sham group, while the addition of sh-SNHG14 reduced levels of above inflammatory factors in serum of I/R-induced rats (all *P* < 0.01, Figures 1(e)–1(g)). We then determined the relative expression of miR-124-3p and MMP2 in renal tissues of I/R-induced rats. We found that the relative expression of miR-124-3p was decreased, and the relative expression of MMP2 was increased in the I/R group compared with those in the sham group (all *P* < 0.01, Figures 1(h) and 1(i)). The transfection of sh-SNHG14 partially reversed I/R-induced downregulation of miR-124-3p and upregulation of MMP2 in renal tissues (all *P* < 0.05, Figures 1(h) and 1(i)).

### 3.2. SNHG14 Was Highly Expressed in H/R-Induced HK-2 Cells, and Its Silence Lightened H/R-Induced Injury of HK-2 Cells

**Figure 2:**
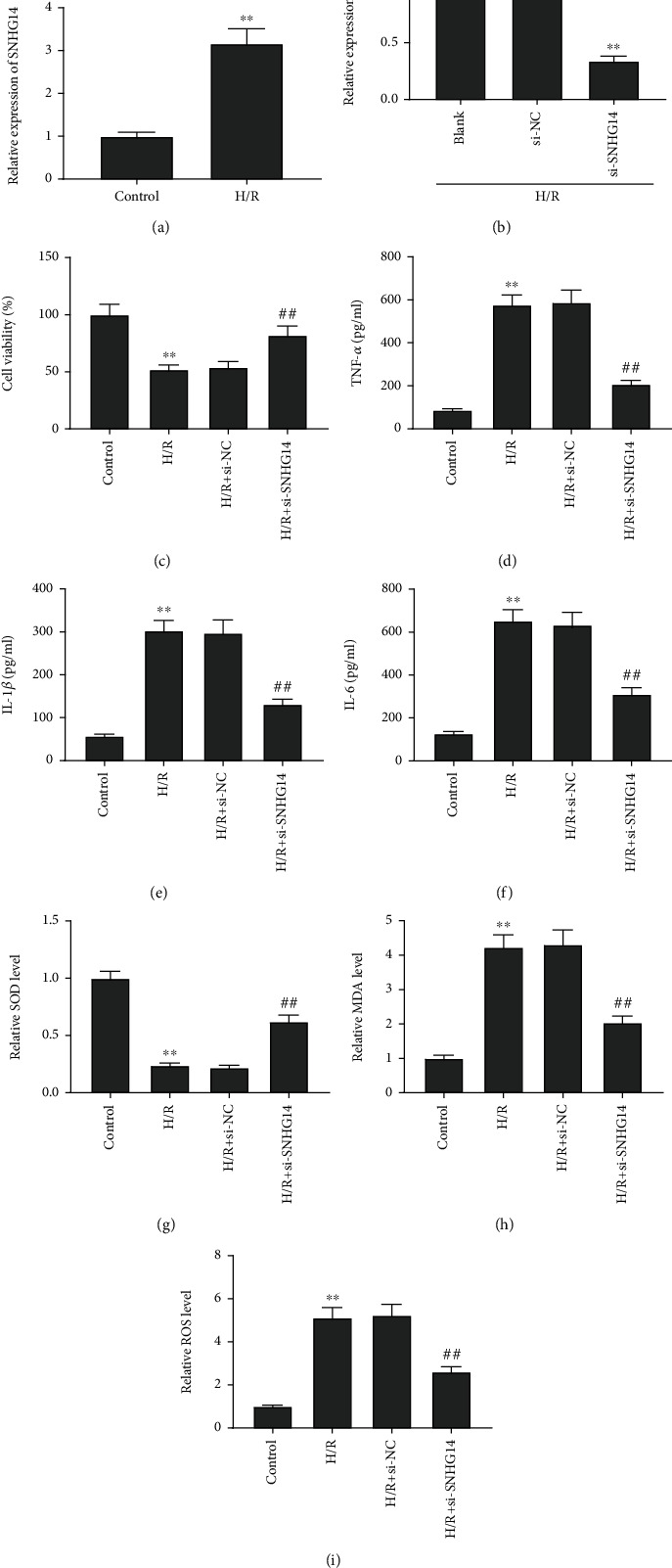
SNHG14 knockdown lightened H/R-induced injury in HK-2 cells. (a) Relative expression of SNHG14. ^∗∗^*P* < 0.01, vs. control. (b) Relative expression of SNHG14. ^∗∗^*P* < 0.01, vs. si-NC. (c) Cell viability. (d) TNF-*α* level. (e) IL-1*β* level. (f) IL-6 level. (g) SOD level. (h) MDA level. (i) ROS level. (C-I) ^∗∗^*P* < 0.01, vs. control. ^##^*P* < 0.01, vs. H/R+si-NC.

Next, we ascertain the expression and role of SNHG14 in HK-2 cells induced by H/R. In line with the outcome shown in I/R-induced rat models, H/R treatment also resulted in a striking increment of SNHG14 expression in HK-2 cells (*P* < 0.01, Figure 2(a)). Meantime, downregulation of SNHG14 in H/R-induced HK-2 cells was observed after transfection of si-SNHG14 (*P* < 0.01, Figure 2(b)). Then, functional experiments were performed. The result of MTT assay revealed that H/R induction led to a decrease of cell viability in HK-2 cells, whereas this decrease was largely counteracted by si-SNHG14 in H/R-stimulated HK-2 cells (all *P* < 0.01, Figure 2(c)). The result of ELISA indicated that levels of IL-1*β*, TNF-*α*, and IL-6 were prominently boosted by H/R induction in HK-2 cells, while evidently reduced by transfection of si-SNHG14 in H/R-stimulated HK-2 cells (all *P* < 0.01, Figures 2(d)–2(f)). Moreover, we found that H/R induction elevated relative levels of MDA and ROS, accompanied by reducing relative level of SOD in HK-2 cells (all *P* < 0.01, Figures 2(g)–2(i)). H/R-mediated increases of MDA and ROS as well as a decrease of SOD were vastly abolished by transfection of si-SNHG14 in H/R-induced HK-2 cells (all *P* < 0.01, Figures 2(g)–2(i)).

### 3.3. SNHG14 Served as a Competing Endogenous RNAs (ceRNA) for miR-124-3p

**Figure 3:**
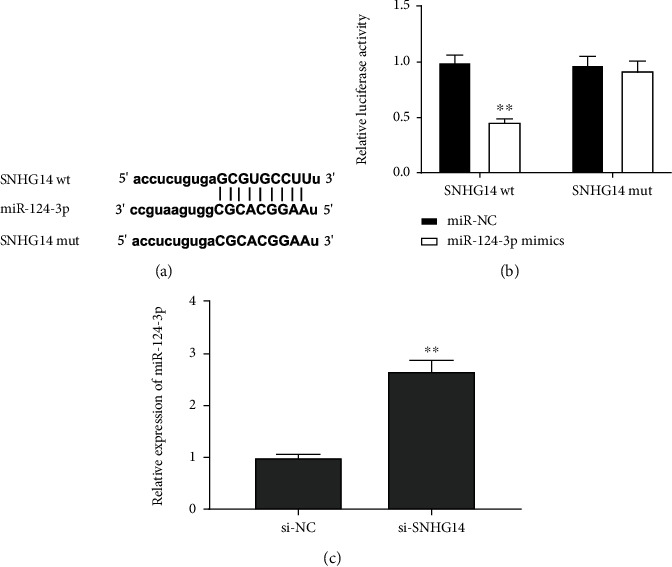
SNHG14 targeted miR-124-3p. (a) The binding sequence between SNHG14 and miR-124-3p. (b) The interaction between SNHG14 and miR-124-3p (DLR assay). ^∗∗^*P* < 0.01, vs. miR-NC. (c) Relative expression of miR-124-3p in H/R-induced HK-2 cells. ^∗∗^*P* < 0.01, vs. si-NC.

In order to expound the regulatory mechanism of SNHG14 silence in H/R-induced HK-2 cells, starbase2.0 was employed to predict targets of SNHG14, and miR-124-3p was discovered to directly target the 3′UTR of SNHG14 (Figure 3(a)). A DLR assay was adopted for confirming this cooperation. The result illustrated that addition of miR-124-3p mimics pronouncedly reduced relative luciferase activity of SNHG14 wt compared with transfection of miR-NC (*P* < 0.01, Figure 3(b)), but there is no notable difference of relative luciferase activity of SNHG14 mut after addition of miR-124-3p mimics in HK-2 cells (Figure 3(b)). Besides, we observed that relative expression of miR-124-3p was remarkably boosted by addition of si-SNHG14 in H/R-stimulated HK-2 cells (*P* < 0.01, Figure 3(c)).

### 3.4. Upregulation of miR-124-3p Alleviated H/R-Caused Damage of HK-2 Cells

**Figure 4:**
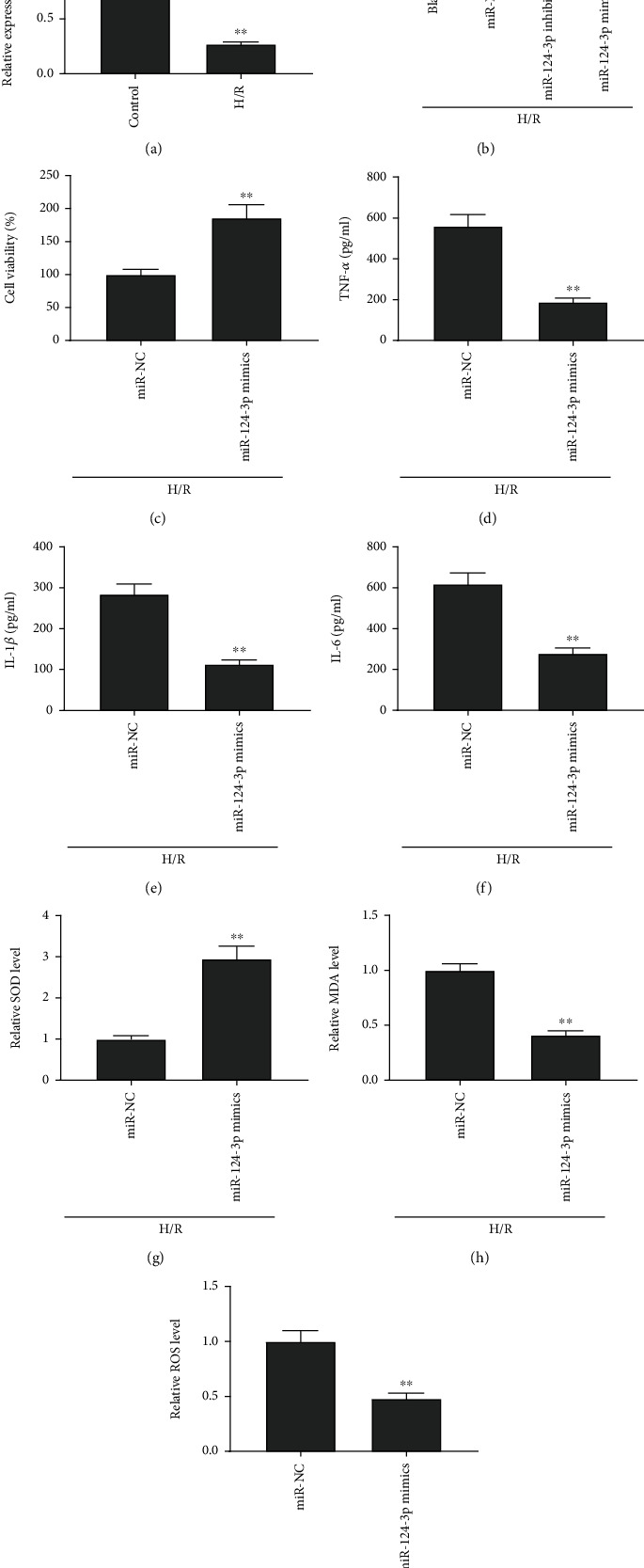
Upregulation of miR-124-3p alleviated H/R-induced injury in HK-2 cells. (a) Relative expression of miR-124-3p. ^∗∗^*P* < 0.01, vs. control. (b) Relative expression of miR-124-3p. (c) Cell viability. (d) TNF-*α* level. (e) IL-1*β* level. F) IL-6 level. (g) SOD level. (h) MDA level. (i) ROS level. (b–i) ^∗∗^*P* < 0.01, vs. miR-NC.

Accordingly, we assessed the expression and function of miR-124-3p in H/R-stimulated HK-2 cells. We discovered that the expression of miR-124-3p in the H/R group was obviously diminished in relative to that in the control group (*P* < 0.01, Figure 4(a)). MiR-124-3p was distinctly decreased by transfection of miR-124-3p inhibitor and strikingly elevated by transfection of miR-124-3p mimics in HK-2 cells induced by H/R (all *P* < 0.01, Figure 4(b)). More importantly, we found that cell viability was enhanced by increasing miR-124-3p in H/R-stimulated HK-2 cells (*P* < 0.01, Figure 4(c)). TNF-*α*, IL-1*β*, and IL-6 were reduced by overexpression of miR-124-3p in H/R-stimulated HK-2 cells (all *P* < 0.01, Figures 4(d)–4(f)). Meantime, upregulation of miR-124-3p reduced levels of ROS and the MDA, whereas elevated the level of SOD in H/R-stimulated HK-2 cells (all *P* < 0.01, Figures 4(g)–4(i)).

### 3.5. MMP2 Was a Direct Target of miR-124-3p

**Figure 5:**
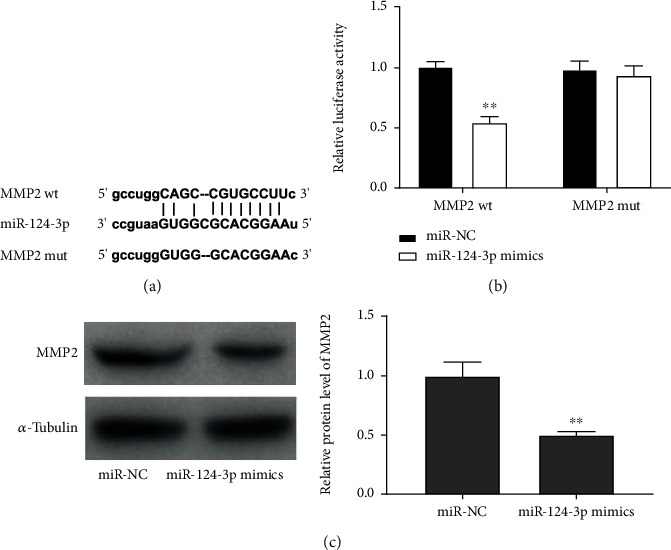
MMP2 is targeted by miR-124-3p. (a) The binding sequence between miR-124-3p and MMP2. (b) The interaction between miR-124-3p and MMP2 (DLR assay). (c) Relative protein level of MMP2 in H/R-induced HK-2 cells. ^∗∗^*P* < 0.01, vs. miR-NC.

Afterwards, we searched for the downstream target of miR-124-3p by using starbase2.0. The prediction analysis denoted that MMP2 was a potential target for miR-124-3p and binding regions between miR-124-3p and MMP2 were observed (Figure 5(a)). The following DLR assay verified that HK-2 cells cotransfected with miR-124-3p mimics and MMP2 wt showed lower luciferase activity than those cotransfected with MMP2 wt and miR-NC (*P* < 0.01, Figure 5(b)). The result of western blot displayed that MMP2 was downregulated by upregulation of miR-124-3p in H/R-stimulated HK-2 cells (*P* < 0.01, Figure 5(c)).

### 3.6. Silencing of SNHG14 Mitigated H/R-Caused Injury via Regulating miR-124-3p/MMP2 Axis in HK-2 Cells

**Figure 6:**
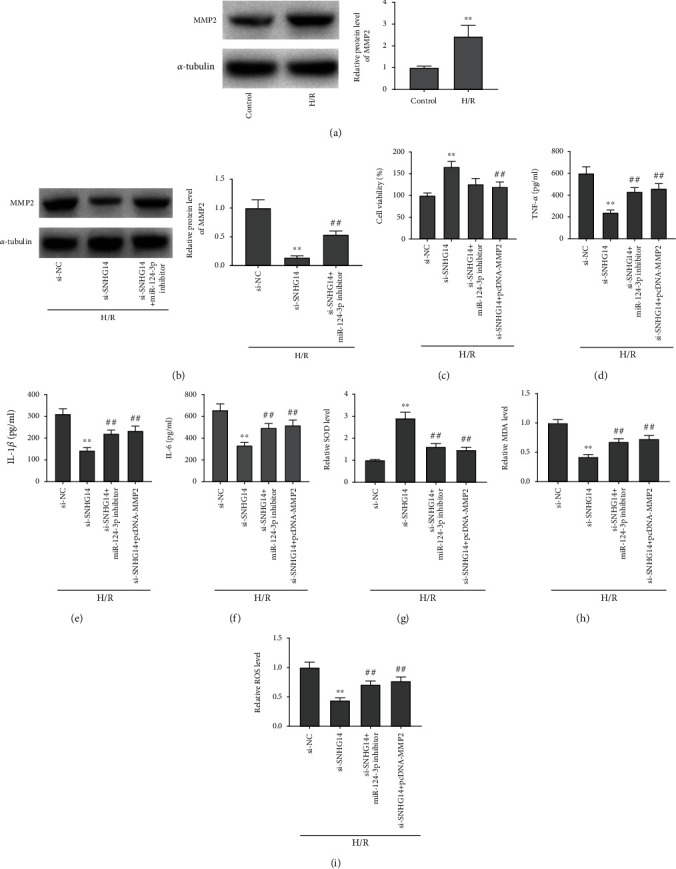
Silencing of SNHG14 mitigated H/R-induced injury via regulating miR-124-3p/MMP2 axis in HK-2 cells. (a) Relative protein level of MMP2. ^∗∗^*P* < 0.01, vs. control. (b) Relative protein level of MMP2. (c) Cell viability. (d) TNF-*α* level. (e) IL-1*β* level. (f) IL-6 level. (g) SOD level. (h) MDA level. (i) ROS level. (b–i) ^∗∗^*P* < 0.01, vs. si-NC.^##^*P* < 0.01, vs. si-SNHG14.

Finally, to certify whether SNHG14 affected H/R-induced HK-2 cells by targeting miR-124-3p/MMP2 axis, the following experiments were conducted. As manifested in Figure 6(a), relative protein level of MMP2 in the H/R group was augmented in comparison with the control group (*P* < 0.01, Figure 6(a)). Silence of SNHG14 reduced relative protein level of MMP2, which was reversed by suppression of miR-124-3p in H/R-induced HK-2 cells (all *P* < 0.01, Figure 6(b)). Furthermore, it was demonstrated that silence of SNHG14 markedly enhanced cell viability, whereas introduction of miR-124-3p inhibitor or pcDNA-MMP2 reversed the enhancement of cell viability caused by silence of SNHG14 in H/R-stimulated HK-2 cells (all *P* < 0.01, Figure 6(c)). Also, silence of SNHG14 markedly reduced levels of TNF-*α*, IL-1*β*, and IL-6, while introduction of miR-124-3p inhibitor or pcDNA-MMP2 reversed reduction of TNF-*α*, IL-1*β*, and IL-6 caused by silence of SNHG14 in H/R-stimulated HK-2 cells (all *P* < 0.01, Figures 6(d)-6(f)). Besides, knockdown of SNHG14 decreased relative levels of ROS as well as MDA and increased the level of SOD, while the suppression impacts of SNHG14 silence on ROS and MDA as well as the promotion impact of SOD were reversed by introduction of miR-124-3p inhibitor or pcDNA-MMP2 in H/R-stimulated HK-2 cells (all *P* < 0.01, Figures 6(g)–6(i)).

## 4. Discussion

AKI refers to is a common complication in hospitalized patients and renal I/R injury is implicated in the pathophysiology of AKI [41, 42]. As reported previously, numerous lncRNAs are upregulated in renal I/R injury [17, 43, 44]. For example, the expression of lncRNA MALAT1 in rats with renal I/R injury is distinctly higher than that in the control group [43]. The expression of lncRNA np_5318 is strikingly elevated in I/R-induced cell models of renal injury [44]. The expression of lncRNA TUG1 is markedly upregulated in renal tissues of I/R-induced rats compared with control group [17]. Consistently, we also observed that lncRNA SNHG14 was upregulated in renal tissues of I/R-induced rats and H/R-induced HK-2 cells as opposed to their controls, implying that SNHG14 might involve in the progression of renal injury.

Convincing evidence has uncovered the importance of SNHG14 in regulation of cell viability and inflammation [22, 45]. Zhong et al. have stated that knockdown of SNHG14 represses inflammation by decreasing levels of IL-6, TNF-*α*, and IL-1*β* and promotes cell viability in oxygen-glucose deprivation and reoxygenation- (OGD/R-) induced cell models [22]. Zhu et al. have reported that silence of SNHG14 reduces production of proinflammatory proteins (IL-6, TNF-*α*, and IL-1*β*) in acute lung injury [45]. Similar to prior reports, we also found that silence of SNHG14 enhanced cell viability and reduced levels of IL-6, TNF-*α*, and IL-1*β* in H/R-stimulated HK-2 cells. At the same time, we discovered that silence of SNHG14 attenuated I/R-induced renal injury in rats and reduced levels of IL-6, TNF-*α*, and IL-1*β* in I/R-induced rats. Moreover, apart from inflammation, oxidative stress is also involved in the pathogenesis of renal I/R injury [46]. Thence, we probed the impact of SNHG14 on oxidative stress and discovered that downregulation of SNHG14 suppressed oxidative stress by decreasing relative levels of MDA and ROS as well as increasing relative level of SOD in H/R-stimulated HK-2 cells. Based on these results, we inferred that downregulation of SNHG14 could mitigate I/R-induced AKI in vivo and in vitro, thereby acting as a promising target for AKI therapy.

In recent years, miRNAs have come into focus as powerful regulators in renal I/R injury, such as miR-21 [47], miR-381 [48], and miR-122 [49]. In particular, miR-124-3p is reported to be abnormally expressed and plays essential roles in modulating the progression of I/R-induced damage [27, 28]. Liang et al. have unveiled that miR-124-3p expression is strikingly decreased in both I/R-induced rat models and cell models, and miR-124-3p reduces the I/R-induced production of TNF-*α*, IL-1*β*, and IL-6 in human cardiac myocytes [27]. Li et al. have pointed out that miR-124-3p is downregulated in I/R-induced rats, and it attenuates I/R-induced nerve injury in spinal cord [28]. Coincident with expression trend of above reports, we also observed that miR-124-3p was downregulated in renal tissues of I/R-induced rats and H/R-stimulated HK-2 cells. Meantime, we discovered that upregulation of miR-124-3p repressed inflammation and oxidative stress as well as enhancing cell viability in H/R-induced HK-2 cells, suggesting that miR-124-3p alleviated H/R-induced renal injury in HK-2 cells. In addition, miRNAs have also been reported to interact with lncRNAs in the pathobiological behaviors of many diseases [50, 51]. Importantly, miR-124-3p has been certified to interact with lncRNA ROR in I/R-induced injury of human cardiac myocytes [27] and interact with lncRNA HOXA11-AS in crystal-induced renal inflammation [52]. Here, we identified miR-124-3p as the direct target of SNHG14, and miR-124-3p was negatively modulated by SNHG14 in I/R-induced rats and H/R-induced HK-2 cells. In the meantime, we discovered that inhibitory effects of SNHG14 silence on inflammation and oxidative stress as well as the promoting effect of SNHG14 silence on cell viability were reversed by suppression of miR-124-3p in H/R-induced HK-2 cells. These data supported the hypothesis that SNHG14 silence mitigated H/R-triggered damage of HK-2 cells via cooperating with miR-124-3p.

Previous documents have uncovered that MMP-2 is upregulated in I/R-induced AKI and engaged in the pathophysiology of I/R-triggered renal injury [17, 53, 54]. Caron et al. have reported that MMP-2 is highly expressed in I/R-induced rat models of AKI [53]. Basile et al. have denoted that MMP-2 expression is elevated in postischemic kidney tissues of rats [54]. Xu et al. have indicated that TUG1 silence lightens inflammation of renal tubular epithelial cell induced by I/R via targeting MMP2 [17]. In line with expression trend of previous literature, we observed that relative mRNA level of MMP2 was increased by H/R treatment and the protein level of MMP2 was boosted by H/R treatment, implying that MMP2 might take part in progression of H/R-triggered renal injury. Moreover, it has been documented that MMP2 can serve as the downstream target of miR-449b-5p in I/R-induced cell models of renal injury [17]. In this study, we certified that MMP2 was targeted by miR-124-3p and inversely modulated by miR-124-3p. According to above findings, we deduced that miR-124-3p could protect against H/R by targeting MMP2 in HK-2 cells. Simultaneously, we found that MMP2 was positively regulated by SNHG14 in I/R-induced rats and H/R-induced HK-2 cells. Importantly, upregulation of MMP2 reversed suppressive impacts of SNHG14 silence on inflammation and oxidative stress as well as the promoting impact of SNHG14 silence on cell viability in HK-2 cells stimulated by H/R. At length, we inferred that silencing of SNHG14 might alleviate I/R-induced AKI by mediating miR-124-3p/MMP2 axis in vitro.

To conclude, our findings for the first time uncovered that SNHG14 was boosted in renal tissues of I/R-induced rats and H/R-stimulated HK-2 cells as opposed to their controls. Knockdown of SNHG14 effectively protected against the damage both in renal tissues of I/R-induced rats and H/R-stimulated HK-2 cells. Overall, this study corroborated that silencing of SNHG14 mitigated H/R-induced injury via competition with miR-124-3p to regulate the expression of MMP2 in HK-2 cells. Altogether, our findings offer insights into the progression of I/R-triggered renal injury involving SNHG14, which acts as a novel target for AKI therapy. However, further investigations are required to illustrate SNHG14/miR-124-3p/MMP2 axis in renal I/R injury in vivo.

## Figures and Tables

**Table 1:** Primers for quantitative real-time polymerase chain reaction (qRT-PCR) in this study.

Gene	Forward	Reverse
SNHG14	5′-GGGTGTTTACGTAGACCAGAACC-3′	5′-CTTCCAAAAGCCTTCTGCCTTAG-3′
MiR-124-3p	5′-ACAGGC TAA GGCTCC CAG TGAA-3′	5′-CGCAGGGTCCGAGGTATTC-3′
MMP2	5′-AGAAGGCTGTGTTCTTTGCAG-3′	5′-AGGCTGGTCAGTGGCTTG-3′
U6	5′-CTCGCTTCGGCAGCACA-3′	5′-AACGCTTCACGAATTTGCGT-3′
GAPDH	5′-CAAGGTCATCCATGACAACTTTG-3′	5′-GTCCACCACCCTGTTGCTGTAG-3′

## Data Availability

All data in the manuscript is available through the responsible corresponding author Shuchi Han.
